# Integrating Ion Mobility Mass Spectrometry with Molecular Modelling to Determine the Architecture of Multiprotein Complexes

**DOI:** 10.1371/journal.pone.0012080

**Published:** 2010-08-10

**Authors:** Argyris Politis, Ah Young Park, Suk-Joon Hyung, Daniel Barsky, Brandon T. Ruotolo, Carol V. Robinson

**Affiliations:** 1 Department of Chemistry, University of Oxford, Oxford, United Kingdom; 2 Department of Chemistry, University of Michigan, Ann Arbor, Michigan, United States of America; 3 Physical and Life Sciences Directorate, Lawrence Livermore National Laboratory, Livermore, California, United States of America; National University of Singapore, Singapore

## Abstract

Current challenges in the field of structural genomics point to the need for new tools and technologies for obtaining structures of macromolecular protein complexes. Here, we present an integrative computational method that uses molecular modelling, ion mobility-mass spectrometry (IM-MS) and incomplete atomic structures, usually from X-ray crystallography, to generate models of the subunit architecture of protein complexes. We begin by analyzing protein complexes using IM-MS, and by taking measurements of both intact complexes and sub-complexes that are generated in solution. We then examine available high resolution structural data and use a suite of computational methods to account for missing residues at the subunit and/or domain level. High-order complexes and sub-complexes are then constructed that conform to distance and connectivity constraints imposed by IM-MS data. We illustrate our method by applying it to multimeric protein complexes within the *Escherichia coli* replisome: the sliding clamp, (β_2_), the γ complex (γ_3_δδ′), the DnaB helicase (DnaB_6_) and the Single-Stranded Binding Protein (SSB_4_).

## Introduction

Multi-protein complexes carry out various critical functions at almost every level of cellular organization, including ion transport, signaling, synthesis, waste management, and cell death [1]. As such, protein complexes comprise some of the most sought-after targets in molecular medicine [2]. However, due to their structural complexity and dynamic character, protein complexes can present a significant challenge to many of the ‘classical’ high-resolution structural biology tools (i.e. X-ray crystallography and Nuclear Magnetic Resonance (NMR) spectroscopy) [3], [4]. Those tools often require large amounts of highly purified samples and many protein complexes are too polydisperse or too scarce within the cellular matrix for structural characterisation. Even when homogeneous proteins can be produced in sufficient quantities, there is often the need to remove dynamic or disordered parts in order to obtain crystals for structure determination. Consequently, certain subunits known to be a constituent of a protein complex are not included in protein databases. Moreover, C- and/or N-terminii of a protein subunit may be truncated to remove dynamic, unstructured regions in order to aid the crystallization process. Therefore, the number of complete high-resolution structures of multi-subunit complexes deposited in structural databases remains relatively low [5].

Over the past few years we and others have been developing the IM-MS technique for multiprotein complexes [6], [7], [8], specifically in our case for elucidating structures of heteromeric proteins [9], [10]. In IM-MS, gaseous ions generated by nano-electrospray ionization (nESI) are separated based on their velocity within a chamber pressurized with inert neutrals. An electric field is applied to pull the ions across the chamber. Larger ions collide more frequently with the neutral gas, hindering their progress and therefore increasing their ‘drift time’ relative to more compact ions [11]. Drift time can be converted to an orientationally-averaged collision cross-section (CCS), which provides information on the overall size and conformation of the ion. Recent research has shown how protein-protein interaction maps, derived from MS data, can be used to generate architectural models for large protein complexes [12], [13], [14]. When combined with homology modelling, such data can be used to produce atomic-resolution models [15]. Recently, the model of 13-subunit eukaryotic initiation factor 3 was refined, using IM data to distinguish two trimeric components within the complex [10]. These results, together with those from other groups [8], [16], [17], [18], [19], have demonstrated the potential of the IM-MS approach for structural biology.

Despite these successes, a number of challenges remain. Chief among these challenges is the computational tools required to derive structural information from the IM-MS data. Similar in many ways to NMR based structure determination, IM-MS data provide a series of distance and connectivity constraints for down-stream modelling. Where NMR structure determination relies mainly on simulated annealing-type molecular dynamics, and other well-developed computational strategies, analogous methods for IM-MS results have only been implemented for peptides and small proteins [20], [21], [22]. In those cases, primary sequence information is input into the simulation, along with any solution-phase structural data, and a low-energy structure is computed for comparison with the experimental CCS value(s). The primary challenge, however, is scaling such approaches to derive structural information for similar data acquired on multiprotein systems. For these large systems, simply entering primary sequence data into a simulation provides too many parameters for optimization on realistic timescales. Moreover, high resolution structural data is often absent, limited or incomplete. A hybrid approach is required, that builds on previous computational approaches, but also accounts for the scale of typical multiprotein complexes. Towards this goal, recent studies have applied a coarse-grained (CG) force field for molecular mechanics analysis of several macromolecular complexes which undergo large conformational changes [23], [24].

Here, we present a computational approach that combines incomplete atomic structures, obtained primarily by crystallographic data, with the experimental constraints derived from IM-MS to generate complete three-dimensional models of multiprotein complexes. This approach relies on two main computational methods, CG and homology modelling, to ‘fill-in’ missing residues from incomplete crystal structures at both the subunit and/or oligomeric level. To build topological models that are in agreement with IM-MS data, multimers are constructed via known archetypal shapes. These tools are applied within a computational workflow designed to build oligomers from smaller building blocks. The method refines each building block using experimental data before an oligomeric complex is constructed and all steps are compared with experimental data.

## Results

### Selection of Systems to Develop the Method

To develop our computational method we focused on IM-MS data and crystal structures for four different protein complexes. First we selected the sliding clamp β_2_ for which 100% of the high-resolution structure data for its dimeric form has been reported [25]. It is responsible for tethering active polymerases to DNA during replication and serves as a starting point for our modelling approach. However, the structures for higher oligomers, including tetramer, hexamer and octamer, observed in our experiments have not been determined previously. Our goal is to reveal the topology of subunits in these oligomeric forms which assemble at high concentrations in solution [26]. We then consider three examples where not only the oligomeric structures are unknown, but there are gaps in the structural information for the building blocks used in the oligomerization process. Each of these examples has a different degree of incompleteness in the available crystal structures. DnaB helicase in *E. coli*, a primary replicative enzyme which coordinates the DNA replication process by opening up the dsDNA, provides an example where a significant portion of its structure is unknown (∼75%). Sufficient homology in relevant databases enables a high-fidelity atomic model of DnaB helicase to be constructed from *E. coli*, based on the corresponding complex form of *Bacillus subtilis* [27]. SSB, a protein responsible for protecting single-stranded DNA during replication, is missing a significant number of residues from its crystal structure (32% of its mass) (PDB ID: 1EQQ) [28]. Even though a more complete structure can be found in protein database, namely 1QVC, the monomers consist of the tetramer within such structure do not share the same fold. Due to the lack of homologous structures, a CG-based approach is used to build topological models of a SSB oligomer observed in our MS experiments. Finally, the γ complex, γ_3_δδ′, contains three different but related proteins but the information provided within the crystal structure of the γ complex for each subunit differs. Specifically, residues not present in the crystal structures are approximately 0.3%, 3% and 13% for δ′, δ, and γ, respectively. In our experiments, four oligomeric species of γ were observed without interaction with other proteins [29]. Here we study the conformational changes observed within the γ complex, compared to oligomeric species of γ in the absence of δδ′, in order to evaluate the accuracy of our method.

### Data Generation and Computational Procedure

**Figure 1:**
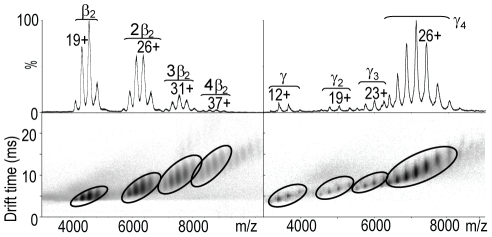
Mass Spectra and ion mobility contour plots superimposed on a common *m/z* scale for the sliding clamp and γ oligomeric species. Data were acquired at a wave height of 8 V for the sliding clamp (left) and 9 V for the γ oligomers (right). The oligomeric species observed and the corresponding charge states are depicted for both proteins.

To begin the model building of the proteins outlined above, we probe the relevant subunits and complexes using IM-MS. Drift time versus m/z contour plots for the sliding clamp, β_2_, the γ subunit and their oligomeric species are shown in Figure 1. For the sliding clamp, charge state series were observed for four distinct species. The major charge state series, centered on an 18^+^ ion, is assigned to β_2_ as the measured mass of 81260 Da is in a close agreement with the calculated mass of 81174 Da. The other charge states series were assigned as 2β_2_, 3β_2_, 4β_2_ with decreasing intensity. By contrast the MS of the γ subunit shows that the major species is a tetramer, centered on a 29^+^ charge state, with lower abundance of ions assigned to monomer, dimer and trimer. IM-MS experiments generate CCS values used in our computational approach for comparison with the calculated CCSs of candidate structures generated *in silico*.

The relatively narrow drift time distributions observed for all oligomeric states (mobility resolution  =  5–8 t/Δt, where t is the drift time and Δt is the width at half-height of the measured distribution) indicate a single conformational family of closely-related structures similar to other protein complex systems described previously [30]. This implies that the overall topology of these proteins are maintained in the gas phase without significant unfolding [30]. To obtain CCSs, drift times are calibrated using protein ions of known CCS [6]. Calibrated CCS values for the β_2_ and γ oligomers are shown in Table S1.

**Figure 2:**
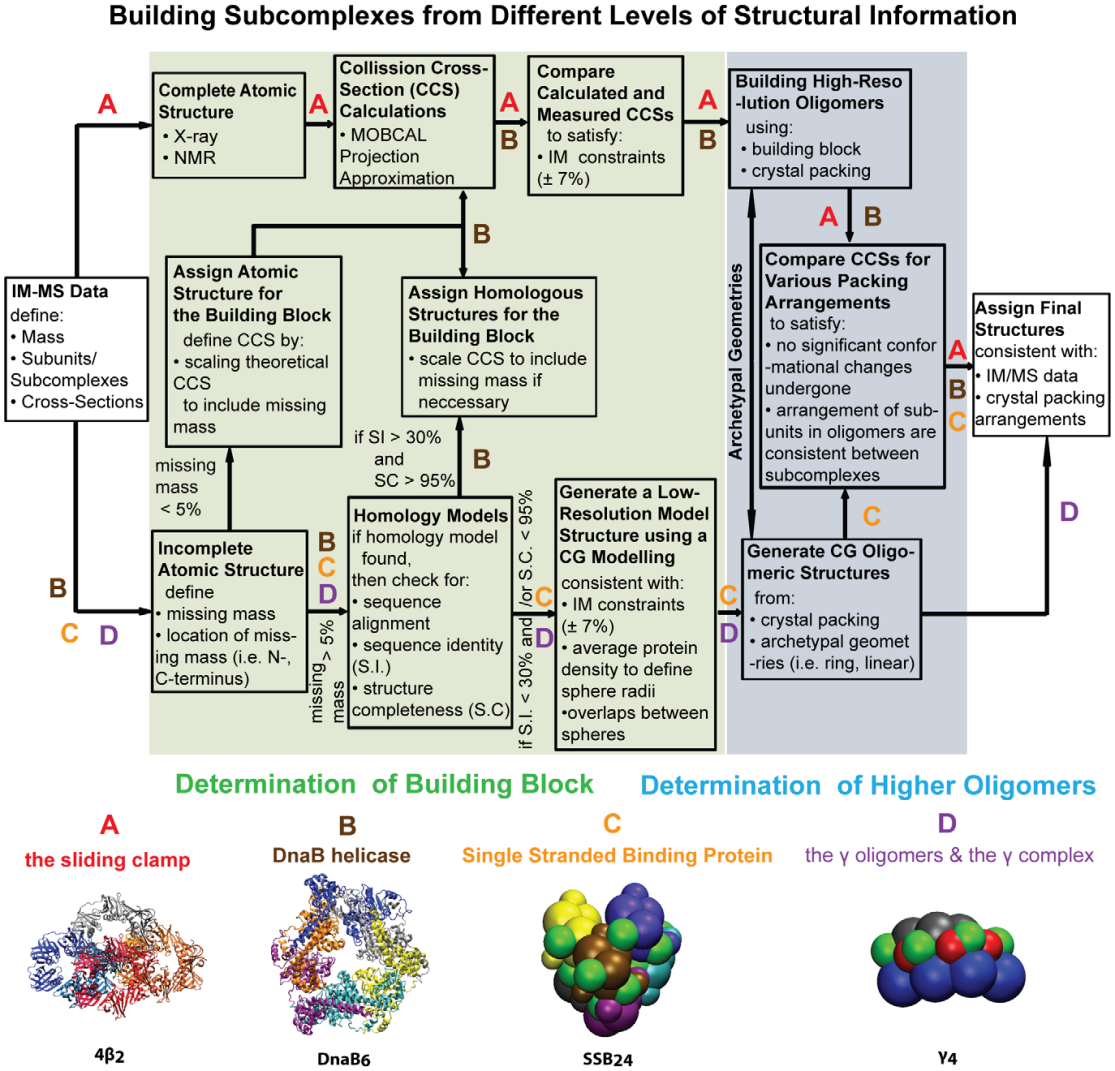
A flowchart of the computational algorithm designed to build multi-protein complexes with different levels of structural data. From the IM/MS data, first the structure of the building block is determined using a variety of computational tools (green shaded). Subsequently, higher-order oligomers are constructed by determining the packing arrangements that provide the best fit to the experimental data (blue shaded). In the first case, two main pathways are suggested based on the structural completeness of the protein under investigation. Starting from a complete high-resolution structure, the building block is assembled, and checked against IM-derived constraints. Incomplete structures are filled by searching for homology in databases enabling atomic models to be constructed. Alternatively, if homology modelling is not feasible, a novel CG approach is used. Higher-order oligomers are built from archetypal geometrical shapes (for low-resolution structures and/or crystal symmetries if high-resolution data is available). The generated packing arrangements are subsequently evaluated with respect to the experimental IM-MS data. The pathways followed for structure determination of the four different protein complexes studied here, as well as their highest-order oligomeric species, are displayed on the flow-diagram.

To reveal the topological arrangements of various *E. coli* replisome complexes, we developed and applied a computational method, an overview of which is depicted as a flow diagram (Figure 2). The first step in our approach is to capture the CCS information from the IM-MS data (Figure 1) and convert these measurements into distance constraints for downstream topology searches. To build model structures successfully, the abundance and quality of IM-MS as well as the available crystallographic data and/or the predicted homologous structures must be sufficient. Therefore, starting from different levels of structural information, we can build high- or low resolution structures of the complete building units, based on the resolution of the data available.

For complete or nearly complete structural information, the building block (e.g., the biological unit for the sliding clamp) is determined upon satisfaction of IM constraints (Figure 2A, β_2_ pathway). This is obtained by comparing the calculated CCS of atomic model structures with the corresponding IM measurements. Large deviations from experimental values indicate potential conformational changes while differences within the experimental error (typically less than 10%) show that the target protein remains compact in the gas phase. On the other hand, starting from incomplete structural data, two different approaches are followed to generate complete candidate structures, based on whether or not a reliable homologous model exists. If a high-fidelity homologous model can be developed, as defined by sequence identity and structural completeness, the algorithmic pathway as described for complete atomic-level structures is followed (Figure 2B, DnaB_6_ pathway). Otherwise, a hybrid approach, which combines high- and low-resolution structural information, is adopted to generate a complete model structure. In this case, the residues absent from atomic coordinates are represented using CG modelling (Figure 2C, pathway for SSB_4_, oligomers of γ subunits and the γ complex).

**Figure 3:**
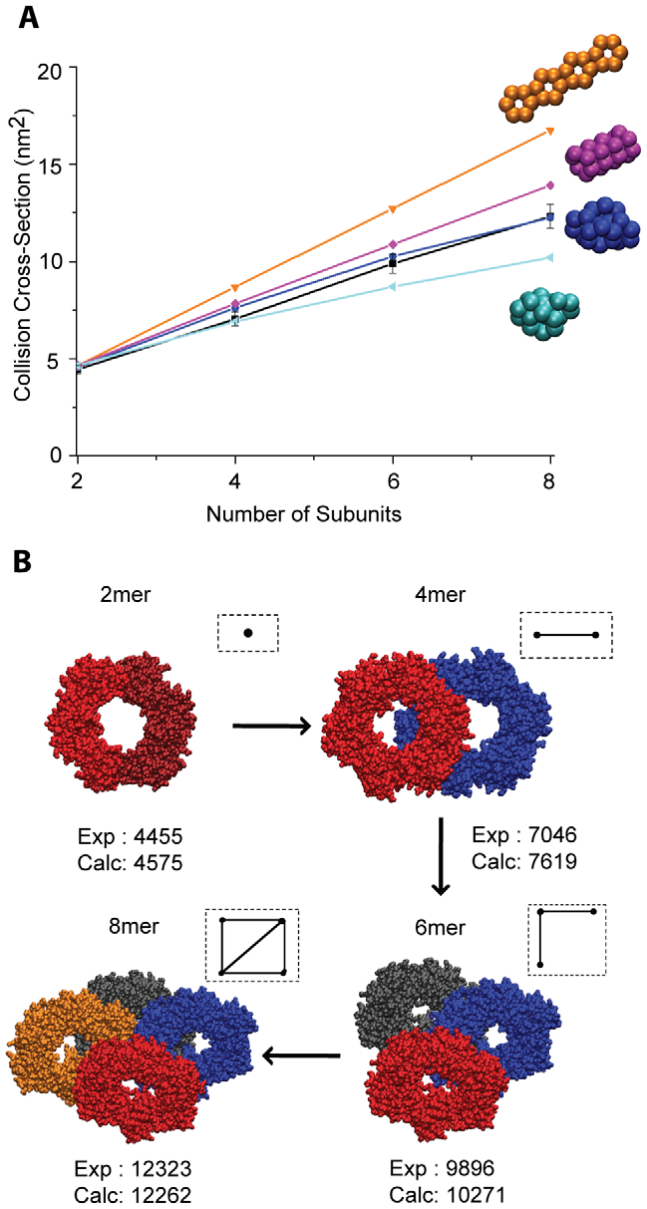
Assembling atomic structures for oligomeric species of the sliding clamp. (A) The CCSs for different archetypal low resolution structures are plotted against the number of subunits and the results are compared with experimental values (black line). The best fit for these values is observed for the compact arrangement mined from the crystal structure (blue). Elongated (orange) and collapsed (cyan) structures are placed at the upper and lower bounds of CCSs, respectively. The edge-to-edge structure (purple) is shown to be in a good agreement with experimental data for oligomers up to the hexamer but it deviates significantly at the level of the octamer. (B) We used the building block for the sliding clamp (β_2_) to construct its oligomeric species. Structural information to build such topologies is mined from crystal symmetries best fitted from (A). Atomic models for tetra-, hexa- and octameric sliding clamps in which the calculated CCSs are in good agreement with experimentally obtained IM data were constructed. A schematic representation of the topological arrangements for each of these oligomers is shown in the inset. The dots represent the center of mass of each dimer and the edges the interconnections between them.

The second part of our method, involves the process of building higher oligomers using either high-resolution or hybrid structures (combined atomic- and CG-level structures) as the building block (Figure 2, highlighted in blue). Higher oligomers are developed using available crystallographic data, structural arrangements mined from crystal packing of symmetrical molecules, and archetypal geometrical shapes (i.e. ring, linear etc). To evaluate candidate models and to assign final topologies of target subcomplexes we examine the various packing arrangements of oligomers with respect to their consistency to IM constraints. Two main assumptions are imposed in our methodology: first, the subunits do not undergo significant conformational changes in forming different complexes, and second the arrangements of subunits in the different oligomers are consistent between sub-complexes. For example, in the case of β_2_ (Figure 3), four different oligomeric forms of the protein (β_2_, 2β_2_, 3β_2_ and 4β_2_) are observed. The models of 4β_2_ should contain the same internal arrangement of subunits as the models of the sliding clamp 3β2 and 2β_2_. If we are successful in building models with these assumptions and that fit the corresponding experimental data, we infer that the assumptions are correct.

### Model Building using atomic structures of complete or nearly-complete subunits

The sliding clamp, β_2_, offers one of the simplest and the most powerful applications for our computational modelling. The crystal structure of the sliding clamp includes the complete structure of β and it is present in PDB as β_2_ (PDB IDs: 2POL and 1MMI) [25], [31]. As such, we measured CCS for β_2_ using IM-MS (Figure 1). Our experimental data shows that β_2_ exist as dimer at low concentrations (1–2 µM). At higher concentrations (>5 µM) oligomeric species (2β_2_, 3β_2_ and 4β_2_) are detected. CCS values range from 4400 Å^2^ for β_2_, to 12300 Å^2^ for 4β_2_ (Table S1). Theoretical CCSs for β_2_, calculated using the Projection Approximation (PA) method employed in MOBCAL, fit well with the measured CCS (Table S1) [32]. This indicates that the structure of the β_2_ is largely maintained within the gas phase.

In order to generate architectures for the sliding clamp oligomers, we used two different approaches. First, we simply built a series of archetypal structures using CG modelling for the representation of the single protomer. This is similar to what has been done previously for ornithine carbomoyl transferase and glutamine synthetase homo-dodecameric complexes [10]. In this case, three overlapping spheres are used to represent the overall architecture for each subunit. This structure is generated using the Shape-Based Coarse-Graining (SBCG) module employed in VMD. This module exploits a neural network algorithm to find the best location for placing the spheres within the atomic coordinates [33]. The radius of each sphere was scaled in such a way that the calculated CCSs for the atomic models agree well with those obtained from their CG counterparts (usually within 1%).

The building block, as determined above, is used to generate different archetypal structures (linear, collapsed, face-to-face stacked, end-to-end stacked) which are, in many cases, inspired by those protein topologies observed in nature for other multi-protein systems. The CCS values of these model structures are then estimated using any one of the available projection approximation-based algorithms for CCS calculation [32], [34]. Calculated and measured CCS values are plotted against the number of β monomers (Figure 3A). The highest CCSs are observed for the linear (Figure 3A, orange) and the lowest for the collapsed (Figure 3A, cyan) models. These two models form an upper and lower bound for our topology search. Good agreement is achieved between the model and the experimental data for multiple trial structures when only small oligomers are considered. At the level of the hexamer and octamer, the only topology that matches the experimental values within error (7%) is that determined through mining the packing arrangements from crystal symmetries (Figure 3A, blue). This appears to best fit the experimental data as a whole, making it the most-likely architecture of the sliding clamp oligomers ranging from dimer to octamer.

Atomic model structures of different oligomeric species are generated from crystal symmetry data. After an exhaustive search of all topologies within such symmetries, various protein packing arrangements are sorted and analyzed according to their systematic agreement with experimental IM-MS data. For β_2_, a set of symmetrical topologies ranging from dimer to octamer is found to be the best match to the experimental data as shown in Figure 3B (on average within 4%). It is also worth noting that such topologies are also the most compact arrangements (found within crystal symmetries) and determined by minimizing the sum of distances between the centers of mass of the building blocks. This arrangement indicates a β_2_ oligomer defined by both ‘face-on’ and ‘side-on’ interactions, forming a series of planar structures in the tetrameric, hexameric and octameric states. A schematic of the topological arrangements for oligomers up to four building blocks are shown in the inset of Figure 3B, where the nodes represent the building blocks (dimer for the sliding clamp) and the edges the interconnections between them.

This example illustrates the potential for our computational method when applied to structural biology problems where the chief goal is to discriminate between two different hypotheses. In this case the two most reasonable structures for β_2_ oligomers conform to either the symmetrical, compact topologies mined from crystal structure (Figure 3A, blue) or the ‘end-on’ stacked structures (Figure 3A, purple), where single monomers within each dimer form the bridging contacts between individual dimers. Compelling arguments can be made for both of these structures, as the driving force behind any β_2_-β_2_ oligomerization is likely to contain a significant electrostatic component due to the highly polar nature of the β dimer. However, our data clearly indicates that only the packing arrangements determined from crystal symmetries match systematically the IM-MS data for the species observed experimentally.

### Building topological models using incomplete X-ray structures

#### DnaB helicase, DnaB_6._

**Figure 4:**
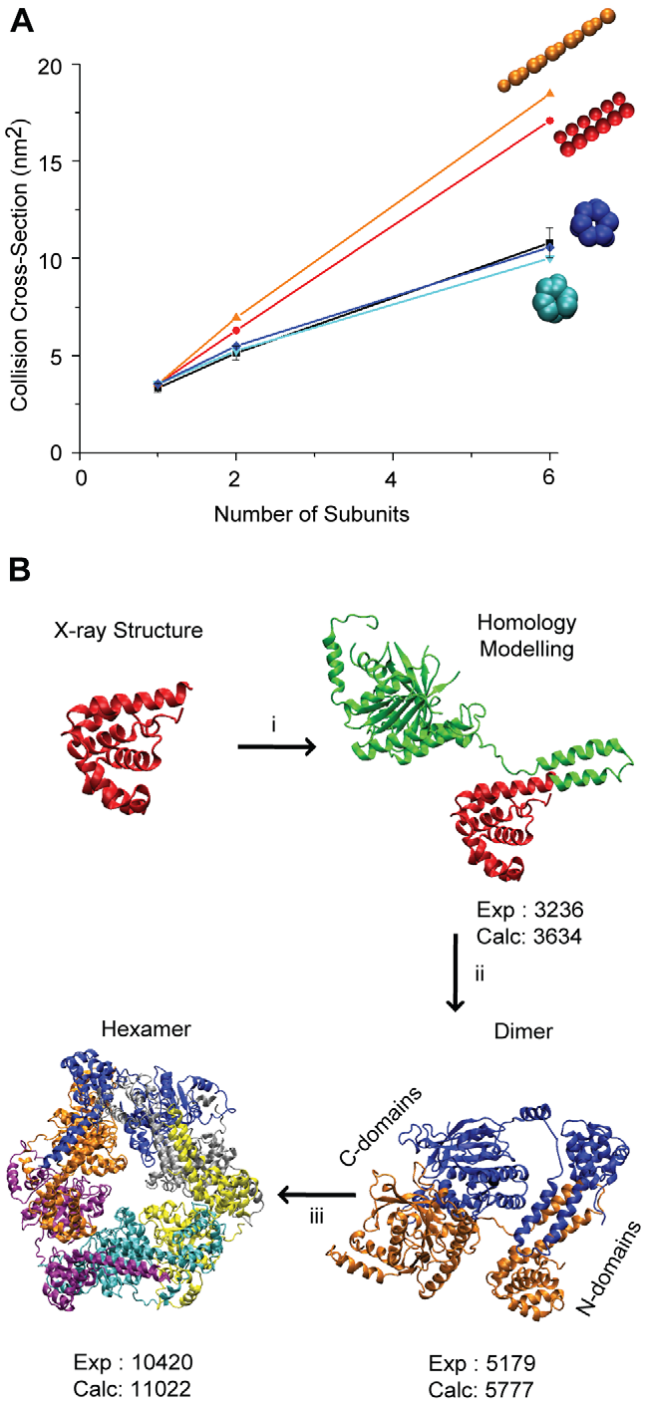
Structural models of DnaB helicase from *E. coli* generated using homology modelling and a CG approach. (A) CCS trend-lines for different CG structures generated from archetypal geometries and the atomic coordinates. These model structures are plotted against the number of subunits and compared with IM measurements (black). The results indicate that the topology determined for the homologous crystal structure, the *E. coli* helicase (PDB ID: 3BGW), shows the best fit to the IM data. (B) a nearly complete homology model of the monomer is built, thus the residues not present in the crystal structure are represented at an atomic-level (in green) (i). The dimeric model is constructed from the monomer by satisfying IM restraints (ii). Utilizing the known hexameric arrangement of the homologue structure (G40P from *Bacillus subtillis*), a double-tiered ring-like structure for hexamer DnaB helicase is generated which is essentially composed of three dimers (iii).

In contrast to β_2_, generating models of DnaB_6_ using a combination of crystallographic and IM-MS data presents an additional challenge: the representation of missing residues from the largely incomplete high resolution structures. Two structures of the N-terminal domain only for the DnaB monomer are available, obtained by crystallography and NMR (PDB ID: 1B79 and 1JWE, respectively) [35], [36]. Here, we used the NMR structure since more residues are present [36]. However, approximately 75% of the protein mass is still absent and the structure of the functional unit (hexamer) of the DnaB is not available (Figure 4A).

Based on our methodology outlined above (Figure 2, pathway C), we searched for a homologous structure in the PDB. The full-length structure of the G40P, the *Bacillus subtilis* bacteriophage helicase, has been recently determined by crystallography and shares 35% sequence identity with the *E. coli* DnaB helicase [27]. Based on the G40P structure (PDB ID: 3BGW), we generated the homologous model of the DnaB monomer using the AS2TS model builder [37]. We then submitted this structure (only the backbone atoms) to the SWCRL program for prediction of side-chain conformations [38]. The homologous structure of DnaB is in good agreement with IM measurements and we use this model as a building block for the oligomeric species (Figure 4A).

To examine a range of archetypal topologies of DnaB_6_, we generated different arrangements similar to the method used for β_2_. Each domain within DnaB monomer is represented by a sphere having the same CCS as the corresponding atomic coordinates. Therefore, based on homologous model for DnaB monomer we build a CG model structure which is subsequently used as a building block for the archetypal geometries shown in Figure 4B. The calculated and measured CCSs are plotted against the number of DnaB subunits. A good agreement is obtained between the model and experiment for multiple trial topologies of dimer (data not shown). However, for DnaB_6_ only one topology satisfies the experimental constraints: the double-tiered ring-like topology (in blue) determined by mining crystallographic data, thus making such topological arrangement the most-likely architecture of this large hexameric helicase.

To build the atomic structure of the dimer of DnaB, we again use the crystal structure for G40P helicase, where all six subunits of G40P are present. In this structure, an unusual assembly mechanism has been revealed, which leads to a unique architecture with dual symmetry: a three-fold N-terminal and a six-fold C-terminal symmetry [27]. Therefore, the hexamer forms a double-tiered ring, where the top-tier contains the N-terminal domains and the bottom tier is composed of the C-terminal domains [27]. The dimer, the building block of the hexamer of G40P, is formed following a cis-trans N-terminal conformation, where the two N-terminal tails come together (see Figure 4B). It is worth noting that a head-to-head dimeric conformation also exists within the crystallized hexameric arrangement [27]. Therefore, we built both models in order to distinguish which is closer to our experimental data. The calculated CCS for the tail- tail dimer was much closer to the measured CCS. This suggests that such dimeric conformation more likely represents the DnaB_2_ structure (Figure 4B). Finally, the hexameric model structure of DnaB is built from the homologous hexameric structure of G40P, as described above. The CCS for such model structure is well within the IM value obtained experimentally (<7%).

#### Single Stranded Binding Protein, SSB_4._

The biological unit of SSB is the tetramer. This is confirmed by our MS data (Figure S1). However, we also observed a 24-mer of SSB (SSB_24_) under conditions of high salt (>1M ammonium acetate) (Figure S1a) which has not been reported previously. In order to determine the architecture of the SSB_24_, first we obtained CCS values using IM-MS and then applied our modelling methodology which in this case follows the pathway C (Figure 2).

**Figure 5:**
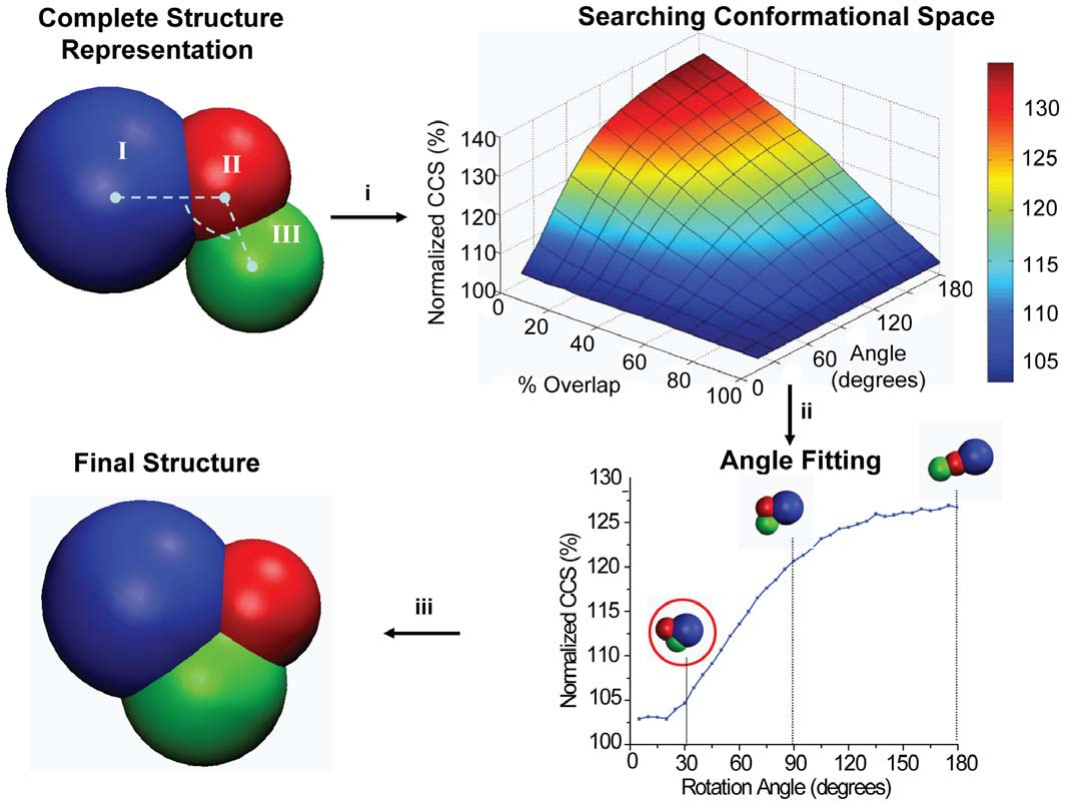
Computational modelling approach for the representation of the incomplete atomic structures using a CG approach: Application on SSB protein. We begin by randomly placing the sphere (shown in green) representing the missing residues within the monomer. Then, the CCSs for all possible locations of such sphere with respect to its overlap with the sphere II and the angle are calculated and normalized to the experimentally obtained CCS value: (i) by applying IM restraints we calculate the best fit for the angle (ii) and we represent the final model structure.

**Figure 6:**
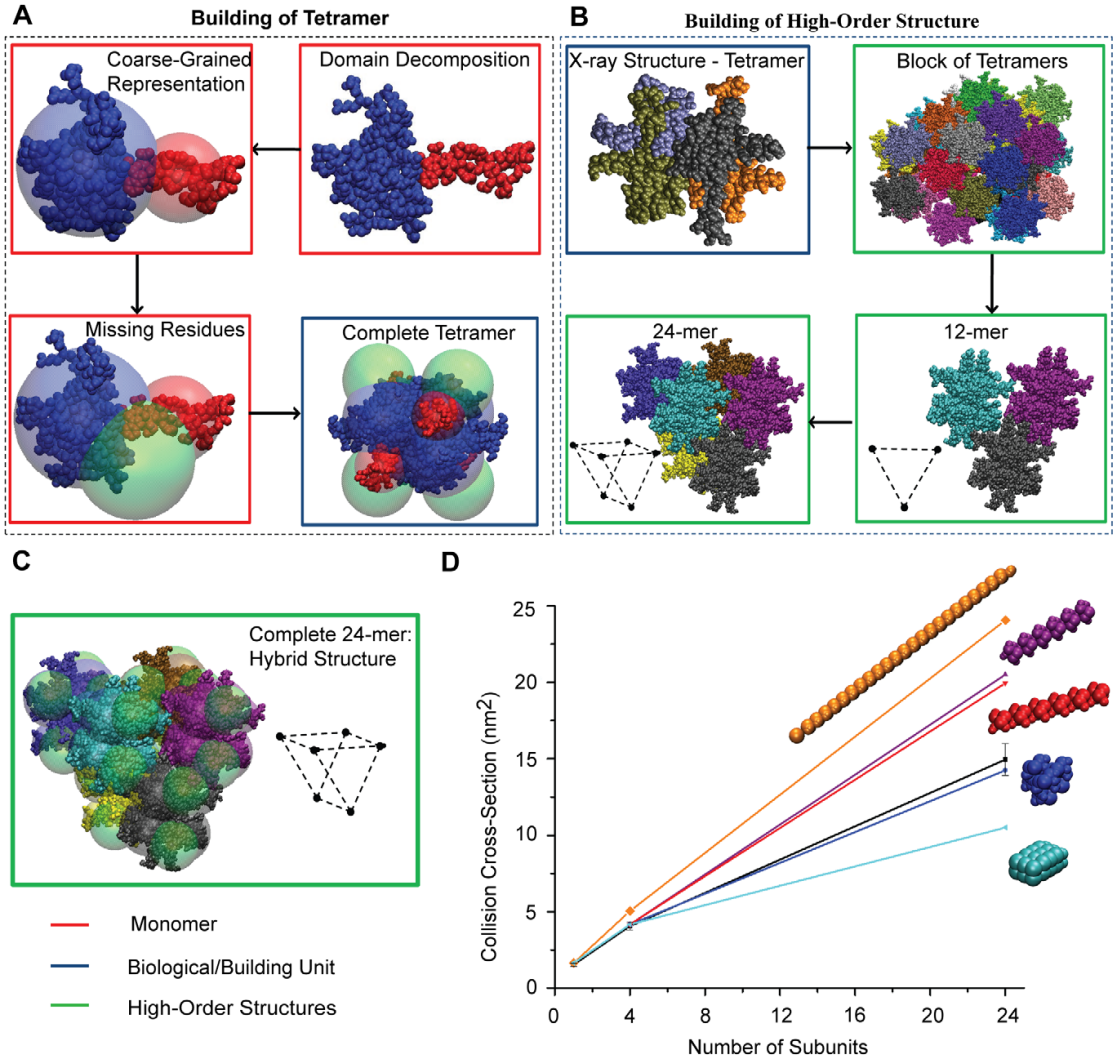
Molecular modelling approach for generating model structures for the SSB protein. (A) The available X-ray structure is decomposed into structural domains using the elastic network model. Each domain is represented by a sphere placed at their center of mass. The size, mass and packing interactions of missing residues within monomers are represented by a sphere docked in the CG structure by fitting the calculated CCS with experimental measurements. The tetrameric SSB is then built using the crystal structure and the full-length CG models. (B) Starting from atomic coordinates, we generate a block of “building units” based on crystal symmetry data. The most compact 12-mer is composed of three symmetrical biological units of SSB and is selected by minimizing the sum of distances between them. Likewise, we built the 24-mer of SSB. (C) We represent the final structure for the 24-mer using the model structure for SSB_4_ and the atomic coordinates mined from crystallographic data. (D) By comparing the CCS trends for different archetypal structures of various SSB oligomers, we show that the compact arrangement (blue) mined from x-ray data provides the best fit with experimental IM measurements (black).

Even though the crystal structure of SSB_4_ is available in the PDB, ∼32% of the mass of the monomer is absent (PDB ID: 1EQQ). Furthermore a reliable homology model for the missing residues is not available. To overcome this deficiency, we applied a CG approach to represent the residues not present in the crystal structure of the SSB monomer (Figure 5). First, the atomic structure of the SSB monomer was decomposed into two structural domains using an Elastic Network Model (ENM) (Figure 6A) [39]. We then represented the crystal structure using two overlapping spheres corresponding to protein domains (blue and red spheres in Figure 6A). The radius of each sphere is defined by the calculated CCS of the atomic coordinates of each domain. The residues that are not present in crystal structure are also represented by a sphere (Figure 5 and 6A, green) where its size is defined by averaging the density of the domains and by calculating the mass for the corresponding residues. The inter-sphere distances between spheres are optimized to fit the IM data (Figure 5). Therefore, using this model of the SSB monomer and the crystal symmetry for SSB_4_, we built a complete model for SSB_4_.

Since no detectable intermediates of SSB_24_ were observed in our experimental data, it is not possible for us to build the SSB_24_ progressively, as we did for 4β_2_ and DnaB_6_. Therefore, it poses additional challenges since fewer experimental constraints are provided for the generation of a high-fidelity model. We begin our structural search by generating archetypal structures similar to the sliding clamp and the DnaB helicase. As expected, the atomic-level search algorithm provides the best fit to all experimental data (Figure 6), with most structures drastically over-estimating the CCS of SSB_24_. Therefore, using the hybrid structure for SSB_4_ (Figure 6A) as a building block, we construct a consensus ‘best’ model for the 24-mer by utilizing crystal symmetries as described above (Figure 6C). The topology of this structure is marked by its stacked architecture, where a planar triangular arrangement of three SSB tetramers form one plane of the complex, and a second similar triangular arrangement stacks on the top, so as to align a small central cavity that runs through the entire structure.

The crystallographic data mining approach that we have adopted provides an excellent screen for likely low-energy multimer configurations of the assembly. On the other hand, crystal growth as a process will not, by definition, explore all possible or likely low-energy configurations of protein oligomers. The CG approach allows us to examine our data using known low-energy configurations, found in nature for other protein complexes, to fully interrogate the topological space for the proteins under investigation. For example, the end-on-end stacked model (red) was inspired by crystallographic symmetry data for SSB octamers. While this configuration was not identified as a likely candidate in the search of crystal structures, the CG strategy allowed us to test the topology against both our ‘best’ model and experimental data for agreement.

### γ Oligomers and the Clamp Loader Assembly

In order to demonstrate application of our IM-MS method to heteromeric complexes we selected the γ complex (the clamp loader complex) from *E. coli*, as our overall aim is to study structural changes within such a complex. The γ complex is composed of three γ subunits, δ and δ′, forming a pentameric ring responsible for loading β_2_ onto DNA during replication [40]. Although the crystal structure of the γ complex is known, large portions of subunits are absent in the structure [41].

Interestingly, the trimer in the γ complex, the γ subunit exists as a monomer-tetramer equilibrium in solution without interacting with other proteins [42], [43]. Our recent studies showed that all four oligomeric species of γ, including monomer, dimer, trimer and tetramer, exist in high salt solution condition [29]. Furthermore, we identified the δ′ subunit as being solely responsible for breaking the γ tetramer into smaller oligomeric species such as γ_3_ and γ_2_, on which δ then associates to form the stable pentameric ring of γ_3_δδ′. Interestingly, we found that δ did not interact even with γ_3_, although all four oligomeric species of γ exist in equilibrium. This led us to speculate that the conformation of γ_3_ may hold the key to δ binding and not the number of subunits. It seems therefore that binding to δ′ confers a specific conformation on γ, priming for binding to δ.

To investigate this, we first compare experimental and theoretical CCSs of the individual subunits (γ, δ and δ′) to identify their conformations. For direct comparison, we account for residues not present in the X-ray structures: approximately 0.3%, 3% and 13% for δ′, δ, and γ, respectively. Measured CCS for δ′ fit well to the calculated values (Table S2). This indicates that the conformation observed in the gas phase is similar to that of the crystal structure, as seen before with other proteins [7], [10], [44]. In contrast, the experimental CCSs for δ or γ were lower by more than 10%, comparing to the theoretical values (Table S2). Interestingly, this implies that δ and γ adopt structures in the gas phase that are significantly more compact than their conformations in the crystal structure of the γ complex, making the comparison between the two more challenging.

**Figure 7:**
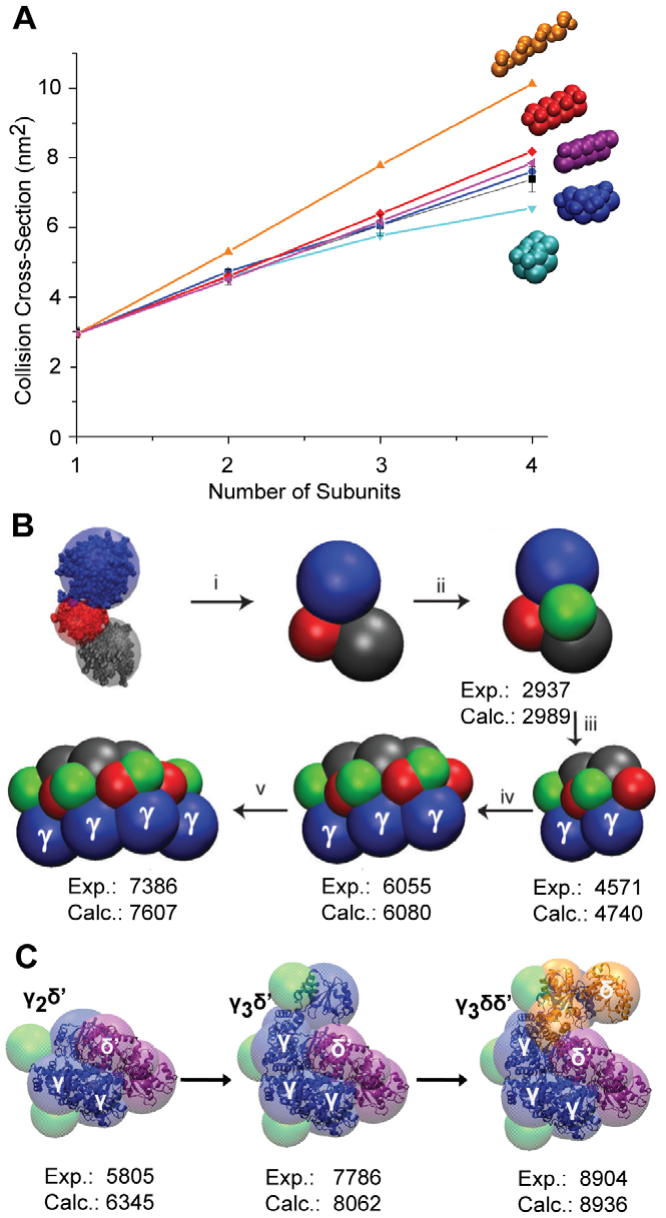
Structural arrangement of the γ complex subunits generated by molecular modelling. (A) CCS trend-lines for various archetypal structure representations. The compact arrangement for the γ_4_ (blue) is in the best agreement with experimental data (black line). (B) Steps for building a CG model structure of the full length γ subunit (i-iii) and higher oligomeric species of γ (iii-v). (i) CG model of the truncated γ based on the crystal structure. (ii) a model structure of the compact form of the truncated γ subunit. (iii) adding the missing residues to the truncated γ to produce the full length γ. Subunit domains I, II, III are represented by blue, red and silver spheres, respectively. The residues not present in the X-ray structure (domain IV) are depicted by a green sphere built by fitting inter-subunit angles (rotation and torsion angles) to the CCS data. Using this model of the γ monomer, CG model structures of γ_2_, γ_3_ and γ_4_ are built systematically (iii-iv). (C) CG model structures for γ_2_δ′, γ_3_δ′ and γ_3_δδ′ subcomplexes.

Similar to the previous examples we investigated the topologies of γ oligomers for various structural classes using simple geometrical modifiers. The trend-lines for the full set of detectable oligomers are compared with experimental values by plotting their theoretical CCSs against the number of subunits (Figure 7A). The results for the compact arrangement, as determined by crystal symmetries (blue), gives the best agreement with the experimental data (black) for all oligomeric species. In contrast, larger deviations from the experimental data were observed for the linear arrangement (red) and edge-to-edge representation (purple). Specifically, for the γ tetramer, the CCSs calculated for linear and edge-to-edge structures are 11% and 6% higher than the experimental measurements, respectively. In contrast, the corresponding value for the compact structure is 3% higher. We also generated model structures for a highly elongated arrangement and a collapsed structure to represent the low and upper bounds in our search.

Based on the results outlined above, we examine the structures of the free γ subunit and its oligomers using CG modelling and the available crystal structures, similar to the method used for the other protein complexes [10], [44]. Since γ and δ share the same fold, a more compact model structure for γ was generated, in line with the crystal structure of δ in the γ complex. Based on the CG model of the γ monomer, generated using spheres to represent the structural domains, we built systematically model structures for higher oligomeric species (γ_2_, γ_3_ and γ_4_) to comply with our experimentally determined CCS values and known γ binding motifs from crystal structure analysis (Figure 7B). Interestingly we found that our model structures of γ became compact for higher oligomeric forms with γ_4_ likely forming a semi-closed ring-like structure.

Finally, to verify the conformational changes that we proposed for γ upon δ′ binding, we studied structures of the γ subunits in the presence of δ′, again by comparing experimental and theoretical CCSs of the γ_2_δ′ and γ_3_δ′ sub-complexes. While keeping the tertiary structures of γ and δ′ as in the crystal structure of the γ complex, we produced CG models of γ_2_δ′ and γ_3_δ′ with the fourth sphere accounting for missing residues (Figure 7C). Calculated CCSs for these models show good agreement with the measured values (Table S2). This suggests that in the presence of δ′, the compact topology observed above for γ in isolation opens, adopting a conformation similar to that in the γ complex. Similarly, theoretical and experimental CCS values for a model structure for γ_3_δδ′ correspond well, implying that the compactness observed within γ and δ alone is no longer evident in the presence of δ′. Overall, therefore, in both cases we have established that δ′ confers conformational changes leading to more open topologies of the γ and δ subunits. These results, therefore, show that the application of experimental CCS measurements and CG atomic modelling can lead to a clear topology assignment for both homo- and hetero- complexes and their intermediate subcomplexes, especially in cases where multiple hypothetical topologies for the complex have already been proposed.

## Discussion

We have shown that by integrating different levels of structural data, ranging from low- (IM-MS) to high-resolution (crystallography and NMR), into a single computational approach, we can reveal the molecular architecture of multiprotein complexes. This was achieved by developing a computational algorithm that uses various modelling tools to generate candidate model structures. Central to this approach are the CCSs used to generate spatial restraints for determining the topological arrangements of subunits within subcomplexes. The model structures of oligomers are generated using “building blocks” determined upon satisfaction of these restraints. This approach was applied to four different protein complexes, each with different degrees of structural information available, and resulted in complete three-dimensional structural models of the proteins and their oligomeric species.

The structural characterization of higher-order oligomers of β_2_, the sliding clamp, reveals that the most-likely packing arrangement for its oligomers is a compact structure which propagates to a symmetrical, rectangular topology for 4β_2_. For DnaB helicase, we were able to assign the tail-to -tail dimer conformation of the DnaB dimer and confirmed that the biological unit of DnaB_6_ forms a hexameric structure which is consistent with the ring-like arrangement of the homologous protein from *Bacillus subtilis.* The absence of reliable homology models together with the lack of intermediate SSB oligomers posed additional challenges in determination of the structure of SSB 24-mer. Therefore, by applying our computational method a 24-meric structure of SSB is proposed which essentially forms a pentagon and that can be decomposed into two identical triangles lying on parallel planes. Finally, by investigating the different conformations of γ in the presence or absence of δδ′, we were able to identify δ′ which stabilizes the active conformation of the γ subunits and then allows δ to form the biologically functional γ complex, γ_3_δδ′.

In summary the computational tools that we have detailed here integrate different levels of structural information. They are designed primarily for the interpretation of collision cross section data. We speculate that these tools will become increasingly important, when combined with growing numbers of protein structures in databases and with improved accuracy and precision of CCS measurements. Overall, we believe that this combination is likely to be particularly important for complexes that pose the greatest challenges to established structural biology approaches: those that are heterogeneous and exist at low levels in multiple oligomeric forms or conformational states. As a consequence of this likely application area, we anticipate that our computational methodology will contribute to the emerging hybrid methods that are now used to define such multi-protein systems [3].

## Materials and Methods

### Preparation of protein complex for MS

Separate subunits, β, DnaB, SSB, γ, δ, and δ′ were overexpressed in *E. coli* and purified as described [45], [46], [47], [48]. For MS, β (10 µL at 4.8 mg/mL) was buffer exchanged into 0.5 M NH_4_OAc at pH 6.9 by using micro biospin 6 columns (BioRad). Individual proteins including DnaB (1.2 mg/mL), SSB (1.1 mg/mL), γ (3.2 mg/mL), δ (3.8 mg/mL) and δ′ (4.0 mg/mL) were buffer exchanged into appropriate NH_4_OAc buffers by using Vivaspin 500 concentrators with 10–50 kDa cut off, depending on the size of the protein (Sartorius, UK). Various concentrations and pH of NH_4_OAc buffers were used for each protein: 0.1 M NH_4_OAc at pH 7.6 containing 0.1 mM ATP and 1 mM Mg(OAc)_2_ for DnaB, 1 M NH_4_OAc at pH 7.6 for SSB, 0.1 M NH_4_OAc at pH 6.9 for γ, 1 M NH_4_OAc at pH 6.9 for δ and 0.5 M NH_4_OAc at pH 6.9 for δ′. For the γ complex and the γδ′ subcomplexes, individual subunits were mixed with the stoichiometry of 3:1:1 and 3:2, respectively and then subsequently buffer exchanged into 0.1 M NH_4_OAc using Vivaspin 500 concentrator with 50 kDa cut off. Concentrations for β, DnaB, SSB, γ, δ, and δ′ were measured spectrophotometrically at 280 nm, using ε_280_  =  15130, 29870, 27880, 20940, 46830 and 60440 M^−1^ cm^−1^, respectively.

### Ion Mobility Mass Spectrometry

IM-MS measurements were carried out on a Synapt HDMS system (Waters Corp., UK) described in detail previously [6]. Typically, 2 µL aliquots of solution were electrosprayed from gold-coated borosilicate capillaries prepared in-house as described [49]. Instrument parameters were typically: capillary voltage, 1.4 kV; cone voltage, 40 V; trap collision energy, 12 V; source temperature, 20 °C; backing pressure, 6 mBar. To optimize IM separation, measurements were recorded at 5 wave heights varied from 7 to 10 V, with the traveling wave velocity maintained at 220 or 240 m.s^−1^, depending on the size of a protein. The IMS cell contained N_2_ at a pressure of 0.5 mBar. CCS values reported are an average of the data recorded over all wave heights. Data presented were acquired with a wave height of 8 V and a wave velocity of 240 m.s^−1^.

### Structure representation of missing residues

The algorithm developed here for modelling the structure of missing residues can be decomposed into the following steps: (i) Using the incomplete crystal structure for the monomer, we calculate the mass of the residues not present in the structure (Expasy Proteomics Server: www.expasy.ch/tools/). (ii) If the missing mass is lower than 5% of the mass of the protein, we scale the theoretically calculated CCS linearly to account for the absent residues. For missing sections that are more than 5% of the subunit mass, we search for available homology models from the relevant databases. (iii) If a homology model exists, we then check for sequence identity (S.I.) between the query and template structures and the completeness of the homologous structure. For S.I. >30% and structure completeness (S.C.) less than 95%, homology modelling is our preferred approach for generating a final structure. Structural completeness stands for the percentage of mass present in the atomic structure available and simultaneously accounts for the overall mass of the missing segments. (iv) If the S.I. <30% and S.C. <95%, then we represent the missing mass using CG modelling where we fill-in the residues not present in crystal structure using a single sphere having the same density as the crystallized segments of the monomeric structure. Although not demonstrated in detail in this work, if the tertiary structure of the missing sequence is either known or can be predicted from other sources, multiple spheres (e.g., a string of beads) can be used to better represent the absent structural elements. The known components of the monomer are also represented as spheres, defined at either the subunit level or the domain level, by decomposing the protein using the atomic coordinates into structural domains using ENM [39], [50]. (v) Finally, we generate all possible CG structures by searching the conformational space of the interacting structural components. The final low-resolution structure is selected by comparing the theoretically calculated CCS with experimental IM-MS data for the protein subunits that compose the complex (see Figure 5).

### Generation of Multimeric Complexes

The process of generating multimeric complexes/subcomplexes is summarized in three main steps. First, the complete model structure for a monomer, as determined following the above algorithmic procedure, is used to generate the model structure of the building block (i.e. dimer for β_2_ and tetramer for SSB) based on the available crystal structure. Then, the CCS for this structure is calculated using an appropriately scaled version of the projection approximation (PA) approach as implemented within MOBCAL [32] and the resulting CCS is compared with experimental CCS values. We note that the PA approach to calculating CCS for model structures will underestimate the actual CCS of the structure, as scattering angle is not taken into account during the calculation. We also note that scaled PA calculations generated for a wide array of proteins and complexes for which ∼100% of the atomic structure is known, agree very well with CCS measurements for those same complexes. This indicates a ‘universal compaction’ of all proteins in the absence of solvent which we treat as a systematic error in our approach in an effort to relate our measurements in the simplest manner possible to high-resolution X-ray or NMR data. While this choice blinds our approach to a certain rearrangements that may occur on the subunit level, it also serves to focus our approach on generating accurate quaternary structure models that can be integrated and compared with data acquired in solution.

The next two steps involve the model-building process of high oligomers and the decision strategy for the candidate structures. First, we make use of the crystal symmetries, readily available from crystallographic data, to generate a “block” of symmetry-related structures. For each subcomplex observed in IM/MS experiments, we identify and assign for comparison with experimental data all possible combinations of structures found within the generated “block”. To avoid redundancy in our searching process, we use the building block in all candidate structures that derived from this process. Prior to submitting the candidate structures for theoretical CCS calculations, we further reduce the number of structures based on whether or not our experimental data involves intermediate subcomplexes. Therefore, the candidate structures are built in a stepwise fashion, thus all subcomplexes share n-1 building blocks with their lower-order structures, where n is the number of blocks in the subcomplex. Finally, we compare the calculated CCSs of the candidate structures with the experimental data and if multiple solutions arise, we select the symmetrical structures as it is known that proteins show a strong tendency for symmetry in complexes.

The computer codes used for generation of model structures within the algorithmic procedure described above will be freely available to the academic society by email contact.

## Supporting Information

Figure S1 Mass spectra and IM contour plots for the detectable SSB oligomeric species i) SSB tetramer and 24-mer and ii) SSB monomer. The plots are superimposed on the same m/z scale. The data we acquired at a wave height of 8V for monomer and 11V for tetramer and 24mer.
(1.54 MB TIF)

Table S1 Measured and calculated CCSs of sliding clamp, DnaB Helicase and Single Stranded Binding Protein (SSB).
(0.03 MB DOC)

Table S2 Measured and theoretically calculated collision cross sections of subunits of the γ-complex.
(0.03 MB DOC)

## References

[1] Alberts B (1998). The cell as a collection of protein machines: preparing the next generation of molecular biologists.. Cell.

[2] Hood L, Heath JR, Phelps ME, Lin B (2004). Systems biology and new technologies enable predictive and preventative medicine.. Science.

[3] Robinson CV, Sali A, Baumeister W (2007). The molecular sociology of the cell.. Nature.

[4] Sali A, Glaeser R, Earnest T, Baumeister W (2003). From words to literature in structural proteomics.. Nature.

[5] Berman HM, Battistuz T, Bhat TN, Bluhm WF, Bourne PE (2002). The Protein Data Bank.. Acta Crystallogr D Biol Crystallogr.

[6] Ruotolo BT, Benesch JL, Sandercock AM, Hyung SJ, Robinson CV (2008). Ion mobility-mass spectrometry analysis of large protein complexes.. Nat Protoc.

[7] van Duijn E, Barendregt A, Synowsky S, Versluis C, Heck AJ (2009). Chaperonin complexes monitored by ion mobility mass spectrometry.. J Am Chem Soc.

[8] Wyttenbach T, Bowers MT (2007). Intermolecular interactions in biomolecular systems examined by mass spectrometry.. Annu Rev Phys Chem.

[9] Leary JA, Schenauer MR, Stefanescu R, Andaya A, Ruotolo BT (2009). Methodology for measuring conformation of solvent-disrupted protein subunits using T-WAVE ion mobility MS: an investigation into eukaryotic initiation factors.. J Am Soc Mass Spectrom.

[10] Pukala TL, Ruotolo BT, Zhou M, Politis A, Stefanescu R (2009). Subunit architecture of multiprotein assemblies determined using restraints from gas-phase measurements.. Structure.

[11] Clemmer DE, Jarrold MF (1997). Ion Mobility Measurements and their Applications to Clusters and Biomolecules. J Mass Spectrom.

[12] Hernandez H, Dziembowski A, Taverner T, Seraphin B, Robinson CV (2006). Subunit architecture of multimeric complexes isolated directly from cells.. EMBO Rep.

[13] Zhou M, Sandercock AM, Fraser CS, Ridlova G, Stephens E (2008). Special Feature: Mass spectrometry reveals modularity and a complete subunit interaction map of the eukaryotic translation factor eIF3.. Proc Natl Acad Sci U S A.

[14] Sharon M, Mao H, Boeri Erba E, Stephens E, Zheng N (2009). Symmetrical modularity of the COP9 signalosome complex suggests its multifunctionality.. Structure.

[15] Taverner T, Hernandez H, Sharon M, Ruotolo BT, Matak-Vinkovic D (2008). Subunit architecture of intact protein complexes from mass spectrometry and homology modeling.. Acc Chem Res.

[16] Baumketner A, Bernstein SL, Wyttenbach T, Lazo ND, Teplow DB (2006). Structure of the 21-30 fragment of amyloid beta-protein.. Protein Sci.

[17] Heck AJ, Van Den Heuvel RH (2004). Investigation of intact protein complexes by mass spectrometry.. Mass Spectrom Rev.

[18] Loo JA, Berhane B, Kaddis CS, Wooding KM, Xie Y (2005). Electrospray ionization mass spectrometry and ion mobility analysis of the 20S proteasome complex.. J Am Soc Mass Spectrom.

[19] Pringle SD, Giles K, Wildgoose JL, Williams JP, Slade SE (2007). An investigation of the mobility separation of some peptide and protein ions using a new hybrid quadrupole/travelling wave IMS/oa-ToF instrument.. Int J Mass Spectrom.

[20] Hoaglund-Hyzer CS, Counterman AE, Clemmer DE (1999). Anhydrous protein ions.. Chem Rev.

[21] Jarrold MF (2000). Peptides and proteins in the vapor phase.. Annu Rev Phys Chem.

[22] Kinnear BS, Hartings MR, Jarrold MF (2002). The energy landscape of unsolvated peptides: helix formation and cold denaturation in Ac-A4G7A4 + H+.. J Am Chem Soc.

[23] Korkut A, Hendrickson WA (2009). A force field for virtual atom molecular mechanics of proteins.. Proc Natl Acad Sci U S A.

[24] Korkut A, Hendrickson WA (2009). Computation of conformational transitions in proteins by virtual atom molecular mechanics as validated in application to adenylate kinase.. Proc Natl Acad Sci U S A.

[25] Oakley AJ, Prosselkov P, Wijffels G, Beck JL, Wilce MC (2003). Flexibility revealed by the 1.85 A crystal structure of the β sliding-clamp subunit of *Escherichia coli* DNA polymerase III.. Acta Crystallogr D Biol Crystallogr.

[26] Lane LA, Ruotolo BT, Robinson CV, Favrin G, Benesch JLP (2009). A Monte Carlo approach for assessing the specificity of protein oligomers observed in nano-electrospray mass spectra.. International Journal of Mass Spectrometry.

[27] Wang G, Klein MG, Tokonzaba E, Zhang Y, Holden LG (2008). The structure of a DnaB-family replicative helicase and its interactions with primase.. Nat Struct Mol Biol.

[28] Matsumoto T, Morimoto Y, Shibata N, Kinebuchi T, Shimamoto N (2000). Roles of functional loops and the C-terminal segment of a single-stranded DNA binding protein elucidated by X-Ray structure analysis.. J Biochem.

[29] Park AY, Jergic S, Politis A, Ruotolo BT, Hirshberg D (2010). A single subunit directs the assembly of the *Escherichia coli* DNA sliding clamp loader.. Structure.

[30] Ruotolo BT, Hyung SJ, Robinson PM, Giles K, Bateman RH (2007). Ion mobility-mass spectrometry reveals long-lived, unfolded intermediates in the dissociation of protein complexes.. Angew Chem Int Ed Engl.

[31] Kong XP, Onrust R, O'Donnell M, Kuriyan J, Stukenberg PT (1992). Three-dimensional structure of the β subunit of *E. coli* DNA polymerase III holoenzyme: a sliding DNA clamp.. Cell.

[32] Mesleh MF, Hunter JM, Shavartsburg AA, Schartz GC, Jarrold MF (1996). Structural Information from ion mobility measurements: Effects of the long-range potential.. J Phys Chem.

[33] Arkhipov A, Freddolino PL, Schulten K (2006). Stability and dynamics of virus capsids described by coarse-grained modeling.. Structure.

[34] Smith DP, Knapman TW, Campuzano I, Malham RW, Berryman JT (2009). Deciphering drift time measurements from travelling wave ion mobility spectrometry-mass spectrometry studies.. Eur J Mass Spectrom (Chichester, Eng).

[35] Fass D, Bogden CE, Berger JM (1999). Crystal structure of the N-terminal domain of the DnaB hexameric helicase.. Structure.

[36] Weigelt J, Brown SE, Miles CS, Dixon NE, Otting G (1999). NMR structure of the N-terminal domain of *E. coli* DnaB helicase: implications for structure rearrangements in the helicase hexamer.. Structure.

[37] Zemla A, Zhou CE, Slezak T, Kuczmarski T, Rama D (2005). AS2TS system for protein structure modeling and analysis.. Nucleic Acids Res.

[38] Bower MJ, Cohen FE, Dunbrack RL (1997). Prediction of protein side-chain rotamers from a backbone-dependent rotamer library: a new homology modeling tool.. J Mol Biol.

[39] Kundu S, Sorensen DC, Phillips GN (2004). Automatic domain decomposition of proteins by a Gaussian Network Model.. Proteins.

[40] O'Donnell M, Kuriyan J (2006). Clamp loaders and replication initiation.. Curr Opin Struct Biol.

[41] Jeruzalmi D, O'Donnell M, Kuriyan J (2001). Crystal structure of the processivity clamp loader γ complex of *E. coli* DNA polymerase III.. Cell.

[42] Dallmann HG, McHenry CS (1995). DnaX complex of *Escherichia coli* DNA polymerase III holoenzyme. Physical characterization of the DnaX subunits and complexes.. J Biol Chem.

[43] Pritchard AE, Dallmann HG, Glover BP, McHenry CS (2000). A novel assembly mechanism for the DNA polymerase III holoenzyme DnaX complex: association of δδ' with DnaX_4_ forms DnaX_3_δδ'.. EMBO J.

[44] Ruotolo BT, Giles K, Campuzano I, Sandercock AM, Bateman RH (2005). Evidence for macromolecular protein rings in the absence of bulk water.. Science.

[45] Marians KJ (1995). Phi X174-type primosomal proteins: purification and assay.. Methods Enzymol.

[46] San Martin MC, Stamford NP, Dammerova N, Dixon NE, Carazo JM (1995). A structural model for the *Escherichia coli* DnaB helicase based on electron microscopy data.. J Struct Biol.

[47] Tanner NA, Hamdan SM, Jergic S, Loscha KV, Schaeffer PM (2008). Single-molecule studies of fork dynamics in *Escherichia coli* DNA replication.. Nat Struct Mol Biol.

[48] Wijffels G, Dalrymple BP, Prosselkov P, Kongsuwan K, Epa VC (2004). Inhibition of protein interactions with the β_2_ sliding clamp of *Escherichia coli* DNA polymerase III by peptides from β_2_-binding proteins.. Biochemistry.

[49] Hernandez H, Robinson CV (2007). Determining the stoichiometry and interactions of macromolecular assemblies from mass spectrometry.. Nature Protocols.

[50] Kundu S, Melton JS, Sorensen DC, Phillips GN (2002). Dynamics of proteins in crystals: comparison of experiment with simple models.. Biophys J.

