# Supplementary figures and images for: Integrating Ion Mobility Mass Spectrometry with Molecular Modelling to Determine the Architecture of Multiprotein Complexes
Source: PLoS One. 2010 Aug 10;5(8):e12080. doi: 10.1371/journal.pone.0012080 (PMC2919415; doi:10.1371/journal.pone.0012080)

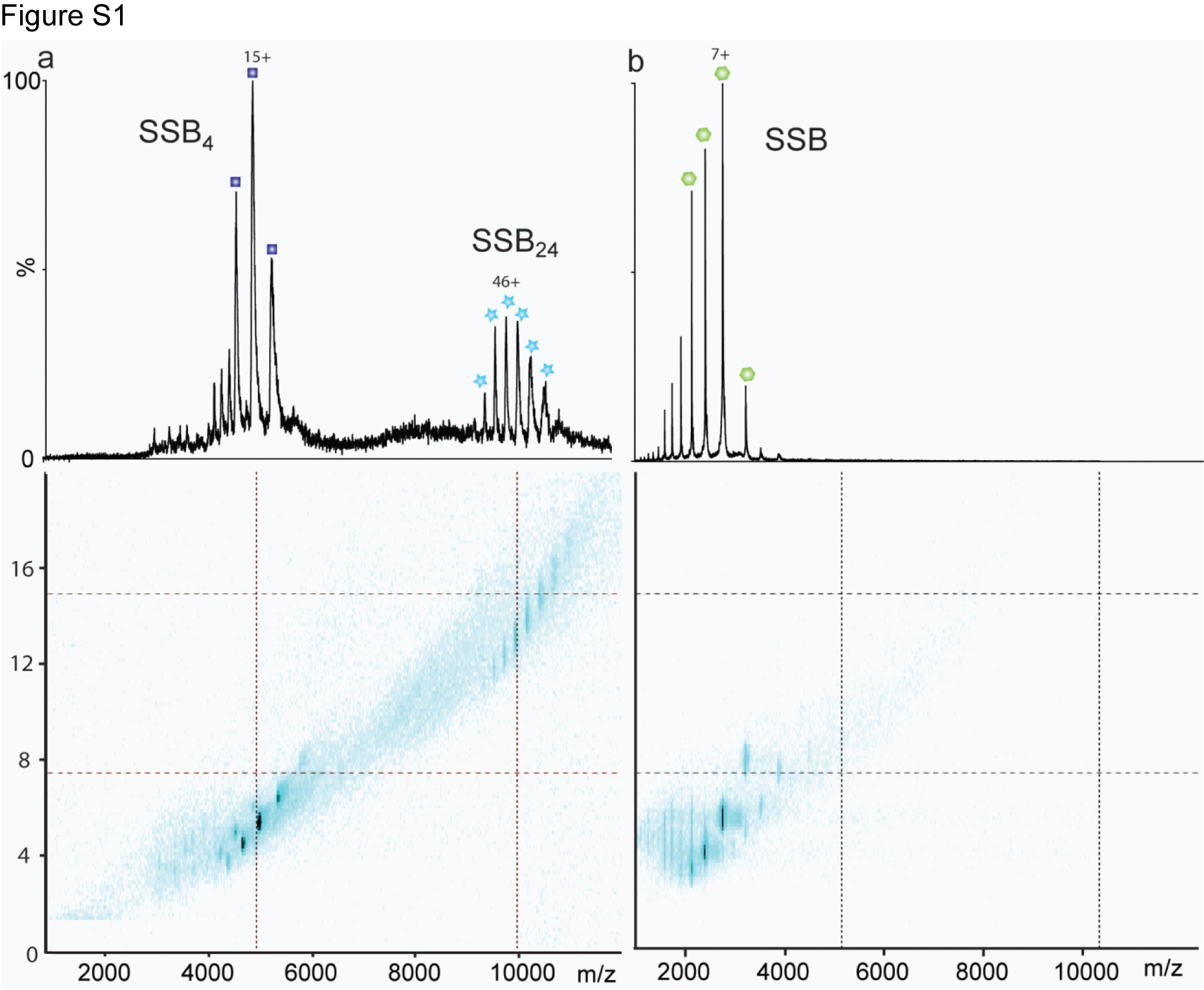

Supplement: Figure S1 — Mass spectra and IM contour plots for the detectable SSB oligomeric species i) SSB tetramer and 24-mer and ii) SSB monomer. The plots are superimposed on the same m/z scale. The data we acquired at a wave height of 8V for monomer and 11V for tetramer and 24mer. (1.54 MB TIF) [file pone.0012080.s001.tif]
