# Supplementary material for: Integrating Ion Mobility Mass Spectrometry with Molecular Modelling to Determine the Architecture of Multiprotein Complexes
Source: PLoS One. 2010 Aug 10;5(8):e12080. doi: 10.1371/journal.pone.0012080 (PMC2919415; doi:10.1371/journal.pone.0012080)
Supplement: Table S1 — Measured and calculated CCSs of sliding clamp, DnaB Helicase and Single Stranded Binding Protein (SSB). (0.03 MB DOC) [file pone.0012080.s002.doc]

**Table S1:** Measured and calculated CCSs of sliding clamp, DnaB Helicase and Single Stranded Binding Protein (SSB).

| Subunits /  sub-complexes | Measured CCS | Calculated CCS  (Atomic model) | Calculated CCS  (CG Model) | Figure |
| --- | --- | --- | --- | --- |
| **Sliding Clamp** | | | | |
| Dimer | 4455 | 4575 | 4654 | 3b |
| Tetramer | 7046 | 7630 | 7739 | 3b |
| Hexamer | 9896 | 10271 | 10415 | 3b |
| Octamer | 12323 | 12262 | 12047 | 3b |
| **DnaB Helicase** | | | | |
| Monomer | 3236 | 3634* | NA | 4b |
| Dimer | 5179 | 5777* | NA | 4b |
| Hexamer | 10420 | 11022* | NA | 4b |
| **Single Stranded Binding Protein** | | | | |
| Tetramer | 4062 | 3379** | 4086 | 6a |
| 24mer | 14941 | 14197** | 14637 | 6b |

* Theoretically calculated CCSs for DnaB helicase are reported after multiplying the atomic coordinate values with a scaling factor of 1.06 based on the percentage of mass missing from both domains.

** The reported CCS calculation for atomic models refers to the truncated sequence (18% missing residues) as seen in 1QVC pdb entry.
