# Supplementary material for: Integrating Ion Mobility Mass Spectrometry with Molecular Modelling to Determine the Architecture of Multiprotein Complexes
Source: PLoS One. 2010 Aug 10;5(8):e12080. doi: 10.1371/journal.pone.0012080 (PMC2919415; doi:10.1371/journal.pone.0012080)
Supplement: Table S2 — Measured and theoretically calculated collision cross sections of subunits of the γ-complex. (0.03 MB DOC) [file pone.0012080.s003.doc]

**Table S2:** Measured and theoretically calculated collision cross sections of subunits of the γ-complex.

| Subunits /  sub-complexes | Measured CCS | Calculated CCS  (Atomic model) | Calculated CCS  (Coarse Grained Model) | Figure |
| --- | --- | --- | --- | --- |
| γ_4_ | 7386 | 7219* | 7607 | 7b |
| γ_3_ | 6055 | 6088 | 6080 | 7b |
| γ_2_ | 4571 | 4568** | 4740 | 7b |
| γ | 2937 | 2934 | 2989 | 7b |
| δ′ | 2565 | 2687 | 2701 | Not shown |
| δ | 2565 | 2825 | 2796 | Not shown |
| γ_2_δ′ | 5805 | 6434 | 6345 | 7c |
| γ_3_δ′ | 7786 | 7113 | 8062 | 7c |
| γ_3_δδ′ | 8936 | 8110 | 8904 | 7c |

* The reported CCS calculation for γ_4_ was performed on all-atom coordinates of symmetrical structures of chains A and B in pdb entry.

** Mean value of CCS calculations based on all-atoms coordinates of chains A,B and A,C from the X-ray structure (PDB ID: 1jr3).
